# Efficacy of acupuncture on menstrual frequency in women with polycystic ovary syndrome: Protocol for a randomized, controlled trial

**DOI:** 10.1097/MD.0000000000008828

**Published:** 2017-11-27

**Authors:** Jing Zhou, Likun Yang, Jinna Yu, Yang Wang, Zhishun Liu

**Affiliations:** a Department of Acupuncture, Guang’anmen Hospital, China Academy of Chinese Medical Sciences; b China Academy of Chinese Medical Sciences, Beijing; c Graduate College of Tianjin University of Traditional Chinese Medicine, Tianjin, China.

**Keywords:** acupuncture, polycystic ovary syndrome, protocol, randomized controlled trial, sham-acupuncture

## Abstract

**Background:**

Acupuncture may improve the menstrual frequency of women with polycystic ovary syndrome (PCOS). However, more sufficient data are needed to improve the efficacy of acupuncture.

**Methods:**

A total of 172 participants diagnosed with PCOS would be randomly assigned to either the acupuncture group or sham-acupuncture group, at a ratio of 1:1. Participants in both groups will receive treatment for 12 weeks, 3 times a week. The primary outcome will be the proportion of participants with at least a 50% increase from baseline in the monthly menstrual frequency from baseline after 12 weeks intervention, while secondary outcomes will be the difference in anthropometrics, serum hormone level, ovarian morphology, anxiety and depression, and quality of life from baseline to after 12 weeks intervention and to 12 weeks postintervention follow-up between groups.

**Discussion:**

The aim of this study is to evaluate the efficacy and safety of acupuncture for improving menstrual frequency and other symptoms of patients with PCOS. The limitation of this trial is that it would be difficult to blind the acupuncturists. In addition, these findings may not be suitable for women with PCOS who are seeking pregnancy.

## 1 Introduction

Polycystic ovary syndrome (PCOS) is a dysfunction of endocrine system in women of reproductive age.^[1]^ Irregular menstruation (oligomenorrhea or amenorrhea), hyperandrogenism, and infertility are the common clinical manifestation of PCOS.^[2]^ The prevalence of PCOS is 6% to 21%, depending on the diagnostic criteria applied and population studied.^[3]^ Approximately 5.6% women in China have this syndrome, according to an epidemiological investigation.^[4]^ The daily life of patients with PCOS would be affected severely due to intensive mental pressure.^[5]^ Moreover, there is also a huge economic burden brought by this disease. A study is reported that 4.36 billion dollars have been spent for evaluating and providing care to reproductive-aged women with PCOS in the United States.^[6]^

As for the diagnosis of PCOS, 3 consensuses have been successively proposed by the National Institutes of Health, by both the European Society for Human Reproduction and Embryology (ESHRE) and the American Society for Reproductive Medicine (ASRM), and by the Androgen Excess Society, respectively.^[7]^ The Rotterdam criteria proposed by ESHRE and ASRM have been recommended to be used in China.^[8]^

Menstrual-related disorders, androgen-related symptoms, and infertility are the common reasons for women with PCOS who visit their doctors. There is no uniform therapeutic regimen of PCOS due to the different goals of treatment. Lifestyle intervention and weight loss should be applied as the first choice for overweight patients.^[9]^ For inducing ovulation, the first-line pharmacological treatment is clomiphene citrate, the second-line pharmacological treatment is the administration of exogenous gonadotropins or laparoscopic ovarian surgery, and the third-line treatment is high-complexity reproduction treatment such as in vitro fertilization or intracytoplasmic sperm injection.^[10]^

Hormonal contraceptives have been recommended to be used as first-line management for the menstrual abnormalities and hirsutism/acne of PCOS. Combined oral contraceptives (COCs) are a combination of low doses of synthetic estrogens and progestogens, which have been used for patients not seeking pregnancy.^[11]^ Major morbidities, including cardiovascular events related to these combined COCs, have been reported among women of reproductive age, although data remain limited for these results.^[12]^

Recently, it has been reported that acupuncture may be useful for improving ovulation rate^[13–15]^ and treating oligo/amenorrhea.^[16]^ A systematic review revealed that there was an improvement in the menstrual frequency of PCOS patients after acupuncture treatment.^[17]^ However, menstrual frequency was not used as the primary outcome, and more sufficient data would be needed to prove the efficacy of acupuncture.^[18,19]^ A sham-controlled randomized trial will be performed in this trial to evaluate the efficacy of acupuncture in improving the menstrual frequency of PCOS patients who do not have fertility requirements.

## 2 Methods

### 2.1 Study aims

In this study, the efficacy of acupuncture on improving the menstrual frequency in women with PCOS will be evaluated.

### 2.2 Study design

**Figure 1:**
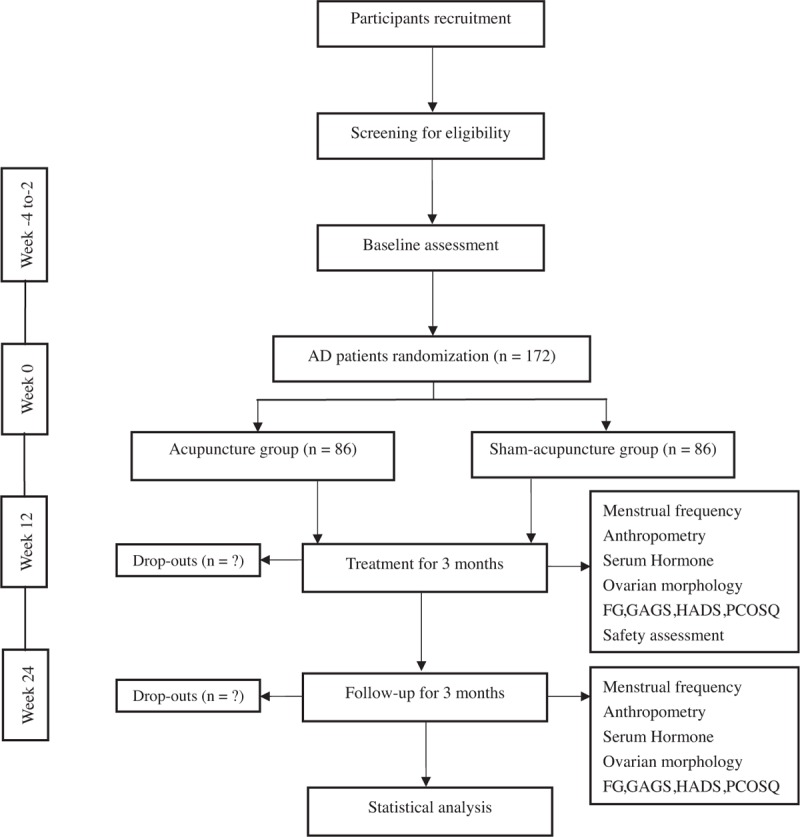
The flow chart of the trial.

**Figure 2:**
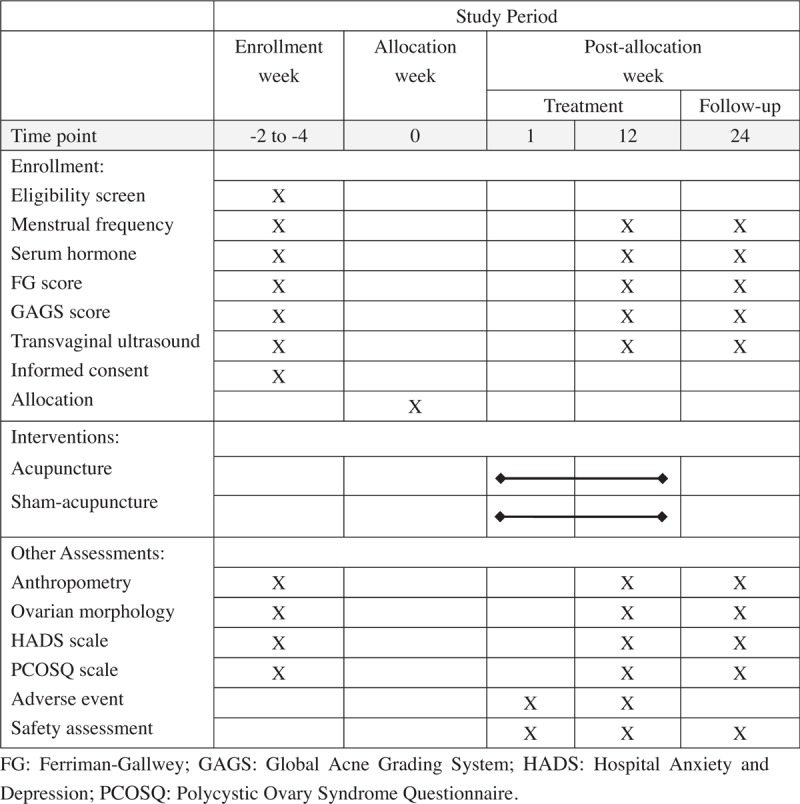
The time point of assessment.

This study is a prospective, randomized and sham-controlled trial. The flow chart is shown in Fig. 1 and the time point of assessment is shown in Fig. 2. The Standard Protocol Items: Recommendations for Interventional Trials (SPIRIT)^[20]^ and the Standards for Reporting Interventions in Clinical Trials of Acupuncture (STRICTA)^[21]^ guidelines were followed during the development of the protocol of this study.

### 2.3 Study setting and recruitment

This study will be conducted between July 2018 and December 2019. A total of 172 participants will be recruited in Guang’anmen Hospital, China Academy of Chinese Medical Sciences, through posters, hospital webs, and networks. The research assistants will be in charge of recruitment, and a gynecologist will be in charge of the diagnosis of the participants.

### 2.4 Inclusion criteria

Participants who fulfill the following criteria will be included.

1. Participants meet the Rotterdam diagnostic criteria^[2]^ with oligomenorrhea or amenorrhea. (Oligomenorrhoea is defined as an intermenstrual interval of >35 days or <8 menstrual bleedings in the past year. Amenorrhea is defined as absent menstrual bleeding or no menstrual bleeding in the previous 90 days.)
2. Participants who have at least 2 of the following features that meet the Rotterdam diagnostic criteria:
  a. Clinical or biochemical hyperandrogenism: Biochemical hyperandrogenemia is defined as a total serum testosterone concentration above normal threshold, and/or clinical hyperandrogenism is defined as a Ferriman–Gallwey (FG) score^[22]^ of ≥5^[23]^ or acne defined by the Global Acne Grading System (GAGS)^[24]^ as “mild”/ “moderate”/ “severe”/ “very severe.”
  b. Polycystic ovary morphology is defined as the presence of ≥12 follicles in each ovary measuring 2 to 9 mm in diameter and/or an ovarian volume >10 mL on transvaginal ultrasound.
3. Participants who are between 18 and 40 years old.
4. Participants who joined the research and provided a signed informed consent voluntarily.

### 2.5 Exclusion criteria

Participants who fulfill any of the following criteria will be excluded.

1. Participants with fertility requirements.
2. Participants with oligomenorrhea or amenorrhea caused by hyperandrogenemia, premature ovarian failure, or hypothalamus or pituitary disorders.
3. Participants with hyperandrogenism caused by congenital adrenal hyperplasia, Cushing syndrome, and androgen-secreting tumors.
4. Participants with endocrine disorders such as thyroid dysfunction, adrenal disorders, hyperprolactinemia, and diabetes mellitus.
5. Participants with severe heart disease, hepatic disease, renal system and hematopoietic system disease, or malnutrition of the whole body.
6. Participants who use hormones or other medications that would affect reproductive function, or received the same protocol of this study in the past 3 months.

### 2.6 Randomization and blinding

#### 2.6.1 Randomization

A 2-week to 4-week baseline assessment will be needed before randomization. The randomization process will be performed by the Clinical Pharmacological Assessment Center at Guang’anmen Hospital using the Statistics Analysis System (SAS) software. The participants will be divided into 2 groups: acupuncture group and sham-acupuncture group; at a ratio of 1:1. Opaque envelopes with sequence numbers marked outside will be used for sealing the paper-written random numbers and grouping information inside. The acupuncturists will obtain the information of the random numbers and group assignment by opening these envelopes.

### 2.7 Blinding

Researchers responsible for the recruitment or data collection, participants, statisticians, and other researchers who are not participants in the treatment process will be blinded.

### 2.8 Intervention

#### 2.8.1 Acupuncture group

Acupuncturists who have an official license and at least 2-year of clinical experience of acupuncture will perform the treatment. Bilateral ST25, EX-CA1, CV4, and SP6 will be selected for treatment. The location of the acupoints will be based on the “Nomenclature and location of acupuncture points”^[25]^ drafted in 2006 by the National Standard of the People's Republic of China (GB/T 12346–2006) and the consensus of experts. After routine sterilization of the local skin, bilateral ST25, EX-CA1, CV4, and SP 6 will be inserted by the needles (0.30 mm in diameter, 40 mm in length) (Hwato Brand, Suzhou Medical Appliance Factory, China) to a depth of 25 to 30 mm to the abdominal muscle layer with the manipulation of lifting, thrusting, and rotating until “de qi” ^[26]^ (the sensation of sourness, numbness, and heaviness). Each session will last for 30 minutes, and the manipulation of lifting, thrusting, and rotating evenly 3 times will be used for CV 4 and SP 6 every 10 minutes. If the date of treatment is during the menstrual circle, the treatment will be continued as usual. Participants will be treated 3 times a week for 12 weeks with 36 sessions.

### 2.9 Sham-acupuncture group

The sham ST25, EX-CA1, CV4, and SP 6, which are 1 cun (25 mm) outward to ST25, EX-CA1, CV4, and SP 6, will be inserted to 2 to 3 mm with needles with a diameter of 0.30 mm and a length of 13 mm. The needles will be inserted without de qi or any manipulation. The treatment sessions will be the same as those in the acupuncture group.

The treatment will be discontinued for pregnant patients. After the treatment, if patients have no menstrual bleeding for 3 months, progesterone (Zhejiang Aisheng Pharmaceutical Co., Ltd., Hangzhou, Zhejiang, China ) will be taken for hormonal withdrawal bleeding. Two 200-mg tablets will be continuously taken every night for 10 days. Patients will stop taking the medicine when menstrual bleeding occurs during these 10 days.

### 2.10 Outcome measure

#### 2.10.1 Primary outcome measure

The proportion of participants who have at least a 50% increase from baseline in monthly menstrual frequency after 3 months of intervention.

Baseline monthly menstrual frequency was calculated through the number of menstrual bleeds in 3 months before intervention divided by 3. Monthly menstrual frequency from baseline to 3 months was calculated through the number of menstrual bleeds during the 3-month intervention divided by 3.

### 2.11 Secondary outcome measure

#### 2.11.1 Change in menstrual frequency

The proportion of participants who have at least a 50% increase from baseline in monthly menstrual frequency to 3 months postintervention follow-up, and the change in monthly menstrual frequency from baseline, to after 3 months of intervention, and 3 months postintervention follow-up.

#### 2.11.2 Change in anthropometry

The change in measurements for height, weight, waist and hip circumference, body mass index (BMI), and waist-hip ratio (WHR) at month 3 and 6. BMI is calculated by dividing the weight by the square of the height. WHR is calculated by dividing the circumference of the narrowest point of the waist by the circumference of the widest point of the hip.

#### 2.11.3 Change in serum hormone level

The change in serum luteinizing hormone (LH), serum follicle-stimulating hormone (FSH), LH/FSH ratio, testosterone (T), estrogen (E), prolactin (PRL), progesterone (Prog), dehydroepiandrosterone (DHEA), sex-hormone binding globulin (SHBG), and androstenedione (AND) from baseline at month 3 and 6. Blood samples will be taken at menstrual cycle days 2 to 4 when ovulation or bleeding occurs. Otherwise, this will be performed on the arbitrary day of the cycle.

#### 2.11.4 Change in ovarian morphology

Calculation changes in bilateral ovaries, mean difference in ovary volume, thickness of the endometrium, and the number of follicles <9 mm by ultrasonography from baseline at month 3 and 6.

#### 2.11.5 Change in hirsutism and acne

Change in FG ^[22]^ and GAGS^[23]^ scores from baseline measured at month 3 and 6. The FG score assesses the hair growth of nine anatomic sites, and has a maximum score of 36 with a scale of 0 to 4 for each site. GAGS evaluate 6 locations, including the forehead, right cheek, left cheek, nose, chin, and chest or upper back. A local score is rated with a factor (1–3 scale) specific to the acne location, multiplying the grade on the acne of this location (0–4 scale). The global score is the summation of all local scores.

#### 2.11.6 Change in anxiety and depression

The score of the Hospital Anxiety and Depression Scale (HADS)^[27]^ changed from baseline at month 3 and 6. HADS is validated and standardized for measuring the state of anxiety and depression.^[28]^ HADS has 2 subscales with 14 items (7 items each), and a total score of 0 to 21 with 0 to 3 for each item. A score of ≥8 shows the presence of anxiety and/or depression.

#### 2.11.7 Change in quality of life (QOL)

The change in Polycystic Ovary Syndrome Questionnaire (PCOSQ)^[5]^ scores from baseline at month 3 and 6. PCOSQ has 26 questions and includes 5 domains, which are emotions, body hair, weight, infertility, and menstrual problems. A lower score indicates a more impaired QOL.^[29]^

#### 2.11.8 Blinding assessment

Participants will answer the following questions after 12 weeks of intervention, in order to assess the blinding: “Do you think you have received traditional acupuncture in the past weeks?” The participants can answer “Yes,” “No,” or “Unclear.”

#### 2.11.9 Expectation value of the acupuncture effect assessment

Participants will answer the following questions before the intervention: “Do you think acupuncture will be effective for treating the disease?” “Do you think acupuncture will be effective for improving the related symptoms of PCOS?” The participants can answer “Yes,” “No,” or “Unclear.”

#### 2.11.10 Safety assessment

All adverse reactions will be presented in tables with a description on the categories, severity, rate of incidence, and correlation with the treatment. Adverse reactions related to acupuncture (severe pain, local hematoma, infection and abscess, and retained needle and broken needle during the treatment), including some discomforts after treatment, will be recorded in time in detail. Adverse events irrelevant with the treatment will also be recorded in detail.

### 2.12 Statistics

#### 2.12.1 Sample size

The calculation of the sample size was based on the primary outcome, which is the proportion of participants with at least a 50% increase from baseline in monthly menstrual frequency during the 12-week intervention.

According to 1 previous study,^[16]^ 38% of participants had increased menstrual frequency after 14 sessions of electro-acupuncture, and the proportion of participants with at least a 50% increase from baseline to 12 weeks was assumed to be 35% in the acupuncture group and 15% in the sham-acupuncture group. Each group sample size of 71 achieves 80% power to detect a difference between group proportions of 20%. A total of 172 participants (86 participants in each group) will be required for the study, with an alpha risk of 5% and a beta risk of 20%, assuming 20% loss to follow-up.

#### 2.12.2 Statistical analysis

Data will be analyzed using SPSS software V.20.0 (IBM SPSS Statistics; IBM Corp, Somers, NY), based on the intention-to-treat (ITT) principle. Missing data will be calculated using the actual observational value instead of the method last observation carried forward. For continuous data, the data will be presented as mean ± standard deviation when normally distributed, or be presented as median (interquartile range) when not normally distributed. Statistical comparisons will be performed by Student *t* test and Wilcoxon rank sum test for continuous data and by *X*^*2*^-test for categorical data. The difference between groups will be analyzed by *X*^*2*^-test or Wilcoxon rank sum test. A *P* value <.05 will be considered statistically significant. The phenotypic distribution will be analyzed according to the included participants, and a subgroup analysis will be conducted according to these phenotypes. Moreover, the phenotypes of these participants will be divided into polycystic ovaries combined with oligo/amenorrhea and hyperandrogenism, oligo/amenorrhea and hyperandrogenism combined with polycystic, and oligo/amenorrhea combined with polycystic ovaries.

#### 2.12.3 Data collection, management, and monitoring

The research assistants will be in charge of the randomization process and data collection. All data will be well-preserved safely, and the double input method will be used for data entry. The data of these participants will be reviewed by the quality monitoring board or revealed with the permission of the principal investigator for emergency circumstances. A quality monitor board consists of a gynecologist, a clinician, an acupuncturist, a medical statistician, and an epidemiologist independent from the researchers and the sponsor. The principal investigator will be in charge of the whole monitoring process. Every detail of adverse events during this study should be recorded in the case report forms. The whole process of this study will be under strict supervision.

#### 2.12.4 Ethics approval and consent to participate

This study will be conducted in accordance with the principles of the Declaration of Helsinki, and has been approved by the Ethics Committee of Guang’anmen Hospital (2015EC15). This trial has been registered at clinical trials.gov (NCT02653911). All eligible participants will be required to provide a sign informed consent before being randomly allocated. These participants will be informed about the aim of the study, the eligible criteria of participants, the course of treatment, the potential benefits, and the risks of the study. All participants will have the right to withdraw from the study at any time. The personal data of these participants will only be used for this study.

#### 2.12.5 Protocol amendment and data dissemination

All modifications of this protocol will be submitted to the Ethics Committee of Guang’anmen Hospital. The data results obtained from this study will be coordinated for dissemination in conferences or peer-reviewed publications.

#### 2.12.6 Study organization and funding

This study will be conducted in and funded by Guang’anmen Hospital, China Academy of Chinese Medical Sciences.

## 3 Discussion

The aim of this study is to evaluate the efficacy and safety of acupuncture for improving menstrual frequency and other symptoms of patients with PCOS. Previous studies have shown that acupuncture can modulate endogenous regulatory systems, including the sympathetic nervous system, endocrine system, and neuroendocrine system.^[30]^ Some studies have also found that acupuncture might be effective for menstrual frequency, improving ovulation rate and serum hormone level.^[31–33]^ Moreover, menstrual irregularity or amenorrhea may increase endometrial proliferation, which is related to potentially premalignant disorders.^[34]^ Therefore, the choice of acupuncture may provide an alternative means for women with PCOS who are not willing to be pregnant, and only want to improve their symptoms. According to a systematic review, no studies have reported the comparison of acupuncture to sham-acupuncture in terms of menstruation frequency at present.^[35]^ In order to reduce the placebo effect of sham-acupuncture, the expectation value of the acupuncture effect will be evaluated before treatment.

Furthermore, assessing the psychological index would be meaningful for the effectiveness evaluation. According to 1 study, women with PCOS had increased anxiety and depression, compared with women without PCOS.^[28]^ A systematic review also reported that PCOS diagnosis is associated with an increased risk of moderate and severe depressive and anxious symptoms.^[36]^ However, very few studies have currently examined the impact of the use of oral contraceptive pills on depressive and anxiety symptoms in women with PCOS.^[37]^ Thus, psychological function should be considered when assessing the effect of treatment.

In addition, PCOS affects the health-related QOL of patients.^[38]^ According to a systematic review, due to the small number of studies, restricted sample size, and the methodological diversity of the questionnaires used, there is a lack of detailed understanding of QOL that patients are facing.^[39]^ Assessing the effectiveness of treatment in terms of QOL should not be ignored. It is reliable and valid to use the Chinese version of PCOSQ for women with PCOS.^[5]^

The limitation of this trial is that it is difficult to blind the acupuncturists. In order to minimize the potential bias, a strict process of randomization and concealment will be performed. Meanwhile, the participants, outcome assessors, and statisticians will be blinded. In addition, our findings may not be suitable for women with PCOS who are seeking pregnancy.

## Acknowledgment

All authors of this manuscript would like to give our sincere thanks to the editors of *Medjaden* Bioscience Limited who have helped us to proofread the manuscript.
